# Sensory afferent segregation in three-eared frogs resemble the dominance columns observed in three-eyed frogs

**DOI:** 10.1038/srep08338

**Published:** 2015-02-09

**Authors:** Karen L. Elliott, Douglas W. Houston, Bernd Fritzsch

**Affiliations:** 1 Department of Biology, University of Iowa, Iowa City, IA, USA

## Abstract

The formation of proper sensory afferent connections during development is essential for brain function. Activity-based competition is believed to drive ocular dominance columns (ODC) in mammals and in experimentally-generated three-eyed frogs. ODC formation is thus a compromise of activity differences between two eyes and similar molecular cues. To gauge the generality of graphical map formation in the brain, we investigated the inner ear projection, known for its well-defined and early segregation of afferents from vestibular and auditory endorgans. In analogy to three eyed-frogs, we generated three-eared frogs to assess to what extent vestibular afferents from two adjacent ears could segregate. Donor ears were transplanted either in the native orientation or rotated by 90 degrees. These manipulations should result in either similar or different induced activity between both ears, respectively. Three-eared frogs with normal orientation showed normal swimming whereas those with a rotated third ear showed aberrant behaviors. Projection studies revealed that only afferents from the rotated ears segregated from those from the native ear within the vestibular nucleus, resembling the ocular dominance columns formed in three-eyed frogs. Vestibular segregation suggests that mechanisms comparable to those operating in the ODC formation of the visual system may act on vestibular projection refinements.

The function of the central nervous system hinges on the specific connection of sensory afferents to provide the brain with information about the environment to react to sensory stimuli. The details of forming such connections are understood down to the molecular level in the visual system^1^ and the olfactory system^2^,^3^,^4^ but are less clear in the inner ear. This deficiency is partly owing to the ear's complexity, with three sensory systems housed in one organ^5^. While the vestibular sensory neurons project partially overlapping gravistatic and angular acceleration information into the vestibular nuclei complex^6^, the spiral ganglion project auditory information to cochlear nuclei. Projection to the cochlear nuclei is possibly specified by transcription factors such as Gata3^5^ and Neurod1^7^. It has been shown that activity fine tunes the temporal pattern of projections to the cochlear nuclei^8^, though the amount of segregation in the cochlear nuclei prior to activity in the cochlea has not been thoroughly investigated at early stages^9^. Based on stereotyped projections prior to hair cell development^10^, it seems likely that inner ear vestibular sensory projections are molecularly targeted to vestibular nuclei^11^ whereas the partial overlapping and partial segregated projection of different sensory organ afferents within the target area^6^,^12^,^13^ may be later driven by differential activity. Activity-mediated partial segregation of overlapping afferents is well known in the visual system as ocular dominance columns (ODCs). ODCs appear either naturally in animals with binocular vision^14^,^15^ or can be induced through grafting of a third eye^16^. It is thought that ODC formation is a consequence of common molecular targeting and differential spontaneous or induced activity^17^,^18^. Indeed, blocking electric activity or synaptic transmission disrupts formation of ODCs^19^, indicating that in the absence of activity the compromise between molecular guidance and activity is shifted toward the molecular cues.

In addition to providing a better understanding of activity-mediated segregation of afferents onto target neurons^18^, transplantation experiments have expanded our understanding of afferent target interaction and selection. In the olfactory system transplanted olfactory afferents transform any brain area they reach into olfactory glomeruli-like organizations^20^,^21^,^22^,^23^,^24^. Misrouted retinal ganglion cell axons of transplanted eyes show a surprising ability to form functional connections, even if they enter the spinal cord^25^. In contrast, inner ear projections into foreign parts of the brain show random distribution with no stereotyped pattern emerging^26^. Afferents of ears transplanted to the orbit grow into various brain areas along the oculomotor, optic, or trigeminal nerves^26^. Afferents entering the midbrain along the oculomotor or optic nerves never reach the vestibular nuclei, indicating absence of long attraction to lure fibers to the vestibular nuclei of the hindbrain. However, afferents reaching the hindbrain along the trigeminal nerve tend to aggregate in the vestibular nuclei through unknown, possibly short-range mechanisms^26^,^27^.

Here we show that a native ear and an ear transplanted in tandem can overlappingly project to the same vestibular nuclei, suggesting an unknown guidance cue that aggregates those fibers preferentially in these nuclei. In contrast to the eye, the ear extracts information about movement and position in space using several distinct organs^6^,^28^. This feature of the ear allows to test whether similar or different sensory stimulation received by identical organs on two adjacent ears can affect the degree of overlap and segregation by simply putting the ears in line or off line with each during grafting. Three-eared frogs with normal ear orientation show normal swimming whereas a rotated third ear results in spinning comparable to the removal of one native ear. We show that two ears grafted in tandem with a similar orientation project largely in an overlapping manner within the vestibular nuclei. In contrast, ears with a 90° rotation with respect to each other result in predominantly segregated afferent projections within the vestibular nuclei. These data suggest that short-range mechanisms likely guide axons to the vestibular nucleus and their final position is likely fine-tuned by activity. Support of initial guidance being molecular comes from the observation of a distinct pattern of innervation of the mouse cochlear and vestibular nuclei prior to the onset of hearing and any known spontaneous activity^10^.

## Results

### Success of transplantation

**Figure 1:**
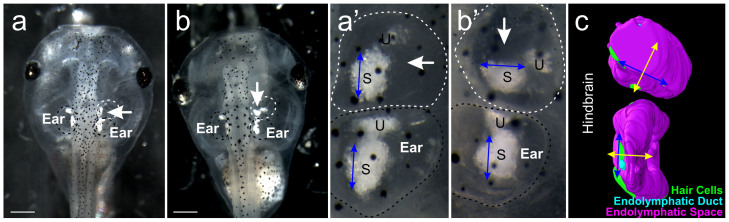
Stage 46 *Xenopus* *laevis* ‘three-eared’ frogs. (a) Embryo with a transplanted third ear in the native orientation. (b) Embryo with a transplanted ear rotated 90 degrees from the native ear. (a’) Higher magnification of the natively-oriented transplanted ear and the right native ear in A. (b’) Higher magnification of the 90 degree rotated transplanted ear and the right native ear in B. (c) Three-dimensional reconstruction of a 90 degree rotated transplanted ear next to the native ear. Endolymphatic space is magenta, endolymphatic duct is cyan, and the hair cells are green. Native, unmanipulated ears are labeled ‘Ear’ and are circled with a black dotted line. Transplanted ears are indicated with a white arrow and are circled with a white dotted line. U, utricle; S, saccule. Blue and yellow arrows indicate ear orientation. Scale bar is 0.5 mm.

Success for ear transplantation was defined as the detection of a third ear, whereas completeness of transplantation was defined as the amount and/or degree of normality of transplanted tissue. Of the 207 successful ear transplantations in which a third ear could be identified, 85% of the ears transplanted contained otoconia (n = 175/207 cases), whereas 14% were just a vesicle and lacked otoconia (n = 29/207 cases). Of the 175 transplanted ears that contained otoconia, 109 were nearly indistinguishable from a normal ear, showing two otoconia-bearing maculae and three semicircular canal cristae. In 52 transplantations, the transplanted ear fused with the native ear, forming a larger ear with multiple sets of otoconia. Fusion occurred more frequently with earlier transplantations (stage 25 vs stage 27). Transplanted ears were not detected in 17 animals. Only animals in which a complete third ear formed (Fig. 1), containing otoconia over the utricle and saccule, and were either in the normal orientation (n = 38) or rotated by 90 degrees (n = 55), were used for further analysis.

### Ear transplantation in a rotated orientation perturbs normal vestibular function in tadpoles

**Figure 2:**
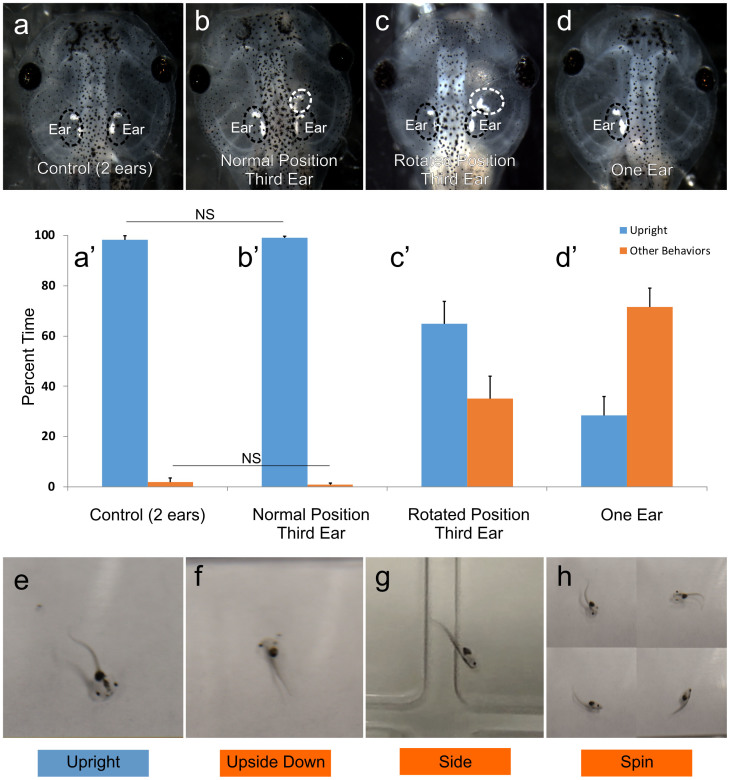
Swimming behavior of normal (two-eared), ‘three-eared’ and ‘one-eared’ frogs. (a–d) Examples of embroys analyzed for their swimming behavior: (a) control, (b) normally-positioned third ear, (c) rotated third ear, and (d) one-eared. (a’–d’) Quantification of percent time spent in various swimming orientations in the first ten seconds of recorded observation for each group of animals: (a’) control (n = 7), (b’)normally-positioned third ear (n = 12), (c’) rotated third ear (n = 11), (d’) one eared (n = 6). Behaviors other than upright swimming (blue) were pooled and referred to as “other behaviors” (orange). All comparisons are significant at p < 0.05 except where noted with horizontal bars and NS, not significant. Error bars reflect the standard error of the means. (e–h) Examples of swimming behaviors observed: (e) upright swimming, (f) upside down, (g) swimming on side, (h) spinning.

Swimming behavior was used as a direct readout of vestibular function in tadpoles. Initial swimming behaviors, normal (swimming upright), swimming on side, swimming in a vertical orientation, swimming upside down, and spinning were determined by observation for animals in which the third ear was in line with the native ear or rotated by 90 degrees (Table 1). Quantification of the duration of various behaviors observed in the first ten seconds of swimming showed that, for animals in which the transplanted ear was in the native orientation (Fig. 2b), most of the time spent swimming is in the upright orientation (Figs. 2b’ and 2e). Slightly less than half of tadpoles with an extra normally-oriented ear (45%) had one additional swimming behavior. Most commonly this was spinning, which was seen for a brief amount of time in 34% of these animals when they were first dropped into the arena. Spinning was immediately followed by re-orientation to upright swimming. This initial spinning behavior was also observed in 35% of control, unmanipulated animals (Fig. 2a), which also spent the majority of the time swimming upright (Fig. 2a’).

In animals in which the transplanted ear was rotated by 90 degrees (Fig. 2c), almost all animals were able to eventually swim upright (93%). Normal upright swimming was the first behavior observed in 31% of these animals. For those able to swim upright, all but two displayed additional swimming behaviors. Additional behaviors included one-sided swimming (49% of animals), upside-down swimming (22% of animals), vertical swimming (42% of animals), and spinning (42% of animals). Quantification of the duration of various behaviors observed in the first ten seconds after being dropped into water shows that animals, in which the transplanted ear was rotated by 90 degrees, spend 35.2 ± 8.9% of the time swimming in orientations other than upright (Figs. 2c’, 2f–2h), which is significantly more time spent than animals in which the transplanted ear was in the native orientation or than control animals [0.8 ± 0.6% and 1.8 ± 1.8%, respectively; p<0.05 (Figs. 2a–2c)]. Though the time spent in orientations other than upright swimming was less for animals in which the transplanted ear was rotated 90 degrees when compared with animals with only one ear (Figs. 2d and 2d’), the overall swimming behaviors were more similar. As with animals with a rotated transplanted ear, most animals with only one ear were able to eventually swim upright (85% of animals). Additional behaviors observed were one-sided swimming (60% of animals), upside-down swimming (25% of animals), vertical swimming (45% of animals), and spinning (90% of animals). Overall, these data show that asymmetrical vestibular input, either having an additional ear in a rotated position, or lacking an ear on one side, affects the swimming behavior of tadpoles whereas symmetric input through two or three similarly oriented ears does not.

### Ear-orientation-dependent sorting of afferent projections to the Hindbrain

**Figure 3:**
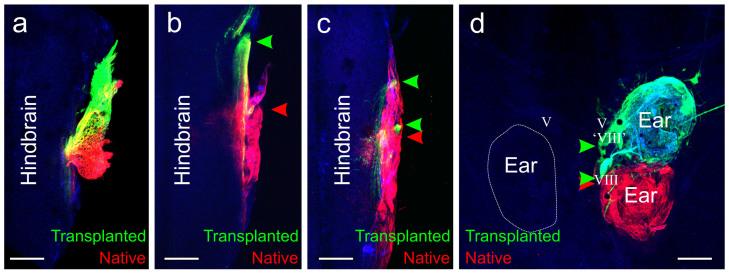
Inner ear afferent projections. (a) Animal in which inner ear afferents projected entirely together in the VIIIth ganglion. (b) Animal in which inner ear afferents from the natively-oriented transplanted ear projected in their own ‘VIIIth’ ganglion and entered the hindbrain separate from the inner ear afferents from the native VIIIth ganglion. (c) Animal in which the inner ear afferents from the 90 degrees rotated transplanted ear entered the hindbrain from both in its own ‘VIIIth’ ganglion and with the native VIIIth ganglion. (d) Animal in which the inner ear afferents leave the natively-oriented transplanted ear both in its own ‘VIIIth’ ganglion and along with the native VIIIth ganglion. Green arrowheads indicate projections from the transplanted ear. Red arrowheads indicate projections from the native ear. V, trigeminal nerve. Scale bar is 100 µm.

Lipophilic dyes of different fluorescence implanted into the native and transplanted ears of fourteen animals successfully labeled afferent projections in the hindbrain. The transplanted ear projected either with the native VIIIth ganglion, completely with its own ‘VIIIth’ ganglion, or in a mixture of both with the native and with their own ‘VIIIth’ ganglion (Fig. 3). In one animal, the transplanted ear, in the native orientation, projected entirely with the native VIIIth ganglion, although neurons from the two ears were mostly segregated within the ganglion (Fig. 3a). In seven animals, the transplanted ear projected with its own ‘VIIIth’ ganglion (Fig. 3b). Five of these animals had rotated ears; two had ears in the native orientation. In the remaining six animals, the transplanted ear partially overlapped with the native ganglion and partially was segregated (Figs. 3c and 3d). Two of these animals had rotated ears; four had ears in the native orientation. Axons from the additional ‘VIIIth’ ganglion from the transplanted ears entered the hindbrain at the approximate level of the trigeminal ganglion, though they did not terminate in the trigeminal nucleus.

**Figure 4:**
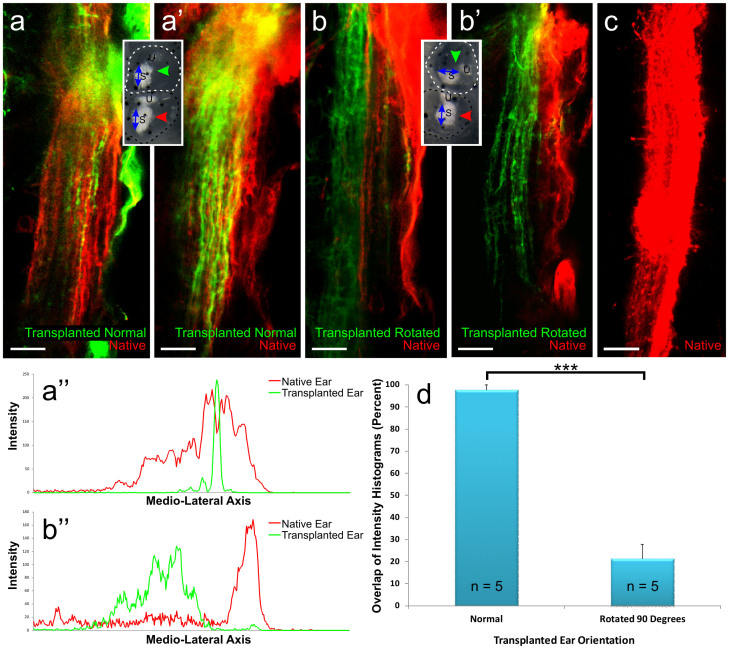
Overlap and segregation of inner ear afferents from transplanted and native ears. (a-a’) Hindbrain from two animals in which the transplanted ear was in the native orientation showing overlap of sensory neurons from the native (red) and transplanted (green) ears. (b-b’) Hindbrain from two animals in which the transplanted ear was rotated by 90 degrees with respect to the native ear showing segregation of sensory neurons from the native (red) and transplanted (green) ears. Insets indicate the transplanted ear orientation. Red and green arrows indicate lipophilic dye placement. (a”) Intensity histogram from an animal with the transplanted ear (green) in line with the native ear (red) shows an overlap of intensity profiles in a single optical section. (b”) Intensity histogram from an animal with the transplanted ear (green) rotated by 90 degrees with respect to the native ear (red) shows a segregation of intensity profiles in a single optical section. (c) Hindbrain showing the native projection when only the control ear was on one side (red). (d) Mean percent overlap and standard error of sensory neurons from the native and transplanted ears for animals in which the transplanted ear was in line with or rotated by 90 degrees with respect to the native ear. 5 animals were analyzed for each condition. Each animal is the mean of measurements taken from 3 different optical sections. Error bars reflect the standard error of the means. ***, p<0.001. Scale bar is 25 µm.

Afferents from all seven embryos with ears transplanted in line with the native ear projected to the same area in the hindbrain with nearly complete overlap of fibers from the two ears (Figs. 4a and 4a’). In contrast afferents from all seven embryos with ears transplanted 90 degrees offset from the native ear projected in general to the same area in the hindbrain, but show either a complete or nearly complete segregation of fibers from the two ears (Figs. 4b and 4b’). Projections into the hindbrain from the rotated ears were always located medial to the native ear projections (Figs. 4b and 4b’), but lateral to the trigeminal tract. For both transplants, in line and rotated, the vestibular nucleus innervated by two ears was broader than the vestibular nucleus innervated by only one ear (Fig. 4c) consistent with previous work showing shrinking after inner ear afferent removal^29^.

The percent overlap of sensory neurons from the native and transplanted ears was calculated from intensity histograms obtained for the two sensory neuron populations (Figs. 4a”–4b”). When the transplanted ears were in line with the native ear, the percent overlap of the narrower histogram with the wider histogram was 97.7 ± 2.3% (n = 5) (Fig. 4d). The range of overlap for a single optical section in these animals was between 66–100%. When the transplanted ears were rotated by 90 degrees with respect to the native ear, the percent overlap of the narrower histogram with the wider histogram was 21.3 ± 6.4 (n = 5) (Fig. 4d). The difference in overlap profiles between animals with normally oriented transplanted ears compared with rotated transplanted ears was significant (p<0.0001). The range of overlap for a single optical section in these animals was between 0–57%. Overall, these data show that vestibular afferents are segregated in a manner dependent on the orientation and presumed differential sensory activity of the ear.

In summary, three eared frogs with normal tandem organization of native and transplanted ear showed near normal swimming behavior and overlapping projections of native and transplanted ear afferents. In contrast, three eared frogs with the transplanted ear rotated around 90 degrees relative to the native ear showed spinning behavior comparable to frogs with one ear removed and displayed near complete segregation of transplanted from native inner ear afferents. Data from mice showing afferent segregation prior to hair cell differentiation and even cell cycle exit of hair cells suggest that initial targeting by inner ear afferents is molecular and is only later fine-tuned through activity.

## Discussion

The results presented here on ‘three-eared’ frogs show that inner ear vestibular afferents of transplanted ears project to the vestibular nucleus when they enter the hindbrain, partially or completely overlapping with the native inner ear projection. We also show different degrees of segregation of native and transplanted ear afferents within the vestibular nuclei depending on the orientation of the transplanted ear. Finally, the transplanted third ear affects swimming behavior of the tadpoles depending on the orientation.

The transplanted ear developed normally in 109 of 207 cases, supporting our previous data in frogs and data from chicks showing future sensory epithelia have already been specified by the otic placode stage^30^,^31^ and continue to differentiate normal after transplantation as previously described in various vertebrates^32^. The fusion of the transplanted ear and native ear may be a side-effect of the early transplantation, as this was observed less frequently when the ear was transplanted at stage 27 rather than at stage 25.

Using lipophilic dyes injected into the native ear and transplanted ear, we showed that, regardless of orientation, both ears project to the same vestibular nuclei of the hindbrain. That afferents from both ears project to and remain confined within the vestibular nuclei region suggests that initial guidance of inner ear sensory afferents is molecular-based, comparable to the targeting of retina ganglion cell (RGC) axons to the midbrain tectum^1^ or lateral line, inner ear and electroreceptive afferents in axolotl^33^. Work in mice has shown that cochlear afferents segregate from vestibular projections prior to the onset of hearing^10^, supporting the idea that initial targeting by inner ear afferents is through molecular guidance^13^,^34^,^35^,^36^. While the exact molecular nature of the guidance remains unknown, the Eph/Ephrin system used by RGCs to guide axons to the tectum/superior colliculus to maintain the visual field might apply to the ear afferents. Ephs and Ephrins have been shown to play some role in axon guidance in the auditory system^35^,^36^,^37^. Given that Ephs and Ephrins are also expressed in vestibular neurons^38^,^39^, suggests that Ephs and Ephrins may play a role in guiding sensory afferents to the proper location centrally in the vestibular nucleus, though this would need to be confirmed by further work.

While sensory neurons from the two ears projected to the same approximate hindbrain region, the precise targeting within the vestibular nucleus depends on the orientation of the transplanted ear with respect to the native ear. The nearly complete overlap in projections from the two ears when the transplanted ear was in the native orientation and the nearly complete segregation of projections from the two ears when the transplanted ear was rotated by 90 degrees suggest that activity-based mechanisms may play a role in fine tuning axon segregation in the vestibular system. The nearly complete segregation of sensory axons in the vestibular nucleus when the transplanted ear was rotated by 90 degrees resembled the ‘ocular dominance columns’ found in the visual system of mammals^14^,^15^ and experimentally induced in ‘three eyed frogs’^16^,^40^,^41^,^42^ as a result of differential activity between two eyes^43^,^44^,^45^,^46^,^47^,^48^,^49^. This activity-based segregation in the visual system likely is a compromise between the common molecular map of both eyes and the differential activity between the two eyes, resulting in a Hebbian sorting of afferents to distinct postsynaptic partners^18^. Extending this insight to the vestibular system, activity may help further segregate axons guided molecularly to vestibular nuclei due to different patterns of activation induced by head movement. Support of axon segregation comes from physiological recordings from second-order neurons in the vestibular nucleus. Recordings show activation by afferents from two different semicircular canals in only 10% of second-order neurons and activation by afferents from all three semicircular canals in only 2% of second-order neurons, whereas activation by afferents from a single semicircular canal occurs in 88% of recordings from second-order neurons^12^.

The ability of ‘three-eared’ frogs to swim normally depended upon the manipulation, which suggests that projections into the vestibular nucleus from the transplanted ear are functional. Animals in which the transplanted ear was in alignment with the native ear swam similar to control animals most of the time. In contrast, animals in which the transplanted ear was rotated by 90 degrees displayed more aberrant swimming, similar to that of animals with only one ear, though not as severe. Given that the latter two manipulations (rotated third ear or only one ear present) results in asymmetrical and mismatched gravitational sensation suggests that bilateral symmetry in sensory epithelia orientation is required for proper sensing of the animals' orientation to proper guide its swimming.

In conclusion, our data imply that mechanisms identified in retina projection development may operate in inner ear projection development: molecular cues and activity combine to wire the ear to vestibular nucleus neurons. Our data imply that evolution of sensory epithelia that acquire different sensory input, such as semicircular canals for angular acceleration perception^28^, or various auditory sensors^50^,^51^, will result in segregated projection allowing for segregated processing of unique information. Continued mutation and selection will eventually result in a segregated projection based on molecular cues^52^.

## Methods

### Ethics Statement

All methods were performed in accordance with the approved guidelines. All animal protocols used in these studies were approved by the Institutional Animal Care and Use Committee at the University of Iowa (#1303056).

### Animals

*Xenopus laevis* embryos were obtained through induced ovulation using injection of human chorionic gonadotropin and fertilized with a sperm suspension in 1X Marc's Modified Ringer's Solution (MMR). Embryos were kept at 18°C in 90 mm Petri dishes containing 0.1X MMR (diluted from 1X MMR, see below) until they reached stage 46^53^.

### Transplantations

Single otic placodes from donor *X. laevis* embryos were transplanted rostral to the native ear in host embryos at stages 25–27. Otic placodes were transplanted either in the native orientation or rotated by 90 degrees. Embryos were kept in 1X MMR for 10–15 min post-transplant to promote healing before being transferred to 0.1X MMR. Healing was confirmed visually as a fusion of the ectoderm above the transplant. Transplants were monitored daily for continued growth. Normal development of multiple otoconia bearing organs of transplanted ear formation was considered to indicate success of transplantations. Only animals with a completely formed transplanted ear, with recognizable features to indicate the orientation of the ear, such as otoconia overlaying the utricle and saccule, were used for further analysis. After embryos reached stage 46, their swimming behavior was assessed (see below). Subsequently animals were anesthetized in 0.02% Benzocaine and fixed in 10% PFA by immersion.

### Swimming Behavior

Animals were dropped into a 3”× 4” swimming arena to observe their initial startle and swimming behavior. All initial behaviors were scored as the following: normal (swimming upright), swimming on side, swimming upside down, swimming vertical, and spinning. Personal observations of all behaviors were recorded for approximately one minute, or until the animal ceased swimming. A sample of animals from each condition were selected and the first ten seconds after they were dropped into the arena were recorded. Percentage of time was calculated for upright swimming and for the other behaviors pooled. Data is reported as means and standard errors of the means. Significance was determined using a two-way ANOVA (Vassar Stats) and post-hoc test (GraphPad). Images for Figure 2 were captured with a Canon T2i camera set at burst recording mode.

### Lipophilic dye labelling

Small pieces of dye-soaked filter paper^54^,^55^ were flattened and implanted into the native (NeuroVue™ Maroon) and transplanted ears (NeuroVue™ Red) in animals previously fixed in 10% PFA. Animals were kept at 36°C for approximately 18 hours to allow for dye diffusion into the hindbrain. Filter paper was removed prior to imaging. Brains were mounted ventral-side up on a slide in glycerol and imaged with a Leica TCS SP5 confocal microscope with a 40x water immersion objective.

### Quantification of sensory neuron overlap

The percent overlap of sensory afferents in the vestibular nucleus of ‘three-eared’ frogs when the transplanted ear was either in the native orientation or rotated by 90 degrees was calculated using the Leica software. Three optical sections were selected for each animal, near the top, middle, and bottom of the image stack. A region of interest line was drawn perpendicularly through the sensory afferents at the approximate midpoint rostral- caudal of the descending tract of the vestibular nucleus. The descending tract of the vestibular nucleus was defined as being outlined by those afferents that projected caudally upon entering the hindbrain. Using the line profile intensity tool, an intensity histogram was provided for the channels representing the two populations of sensory neurons, belonging to the native and transplanted ear. The medial and lateral boundaries of the two histograms were determined manually by using the boundaries generated from a threshold omitting the bottom 25% of the relative intensity. Animals in which either of the fluorescent signals was weak, making it difficult to generate an accurate intensity profile, were not included in the analysis. The percent overlap was calculated by determining the percent of the narrower histogram contained within the wider histogram. The percent overlap obtained for each of the three optical sections was pooled to provide a percent overlap per animal. Animals were pooled to provide the mean percent overlap for the two orientations of ‘three-eared’ frogs. The means and standard errors were calculated. Student's t-test was used as a test for significance.

## Figures and Tables

**Table 1:** Analysis of tadpole swimming behavior

	Swimming Behavior				
Animal Treatment	Upright	Side	Vertical	Upside Down	Spin
**Control (n = 28)**	28	1	1	0	10
**Normal Third Ear (n = 38)**	38	4	1	3	13
**Rotated Third Ear (n = 55)**	51	27	12	23	23
**One Ear (n = 20)**	17	12	5	9	18

Numbers in parenthesis are the total number of animals for each condition.

Numbers represent total animals observed for each swimming behaviors. Some animals displayed multiple behaviors and are included in each total.
